# Higher Stomatal Density Improves Photosynthetic Induction and Biomass Production in Arabidopsis Under Fluctuating Light

**DOI:** 10.3389/fpls.2020.589603

**Published:** 2020-10-21

**Authors:** Kazuma Sakoda, Wataru Yamori, Tomoo Shimada, Shigeo S. Sugano, Ikuko Hara-Nishimura, Yu Tanaka

**Affiliations:** ^1^Graduate School of Agricultural and Life Sciences, The University of Tokyo, Nishitokyo, Japan; ^2^Japan Society for the Promotion of Science, Tokyo, Japan; ^3^Graduate School of Science, Kyoto University, Kyoto, Japan; ^4^National Institute of Advanced Industrial Science and Technology (AIST), Tsukuba, Japan; ^5^Faculty of Science and Engineering, Konan University, Kobe, Japan; ^6^Graduate School of Agriculture, Kyoto University, Kyoto, Japan; ^7^JST, PRESTO, Kyoto, Japan

**Keywords:** leaf photosynthesis, fluctuating light, photosynthetic induction, stomata, stomatal density and conductance, water use efficiency

## Abstract

Stomatal density (*SD*) is closely associated with photosynthetic and growth characteristics in plants. In the field, light intensity can fluctuate drastically within a day. The objective of the present study is to examine how higher *SD* affects stomatal conductance (*g*_*s*_) and CO_2_ assimilation rate (*A*) dynamics, biomass production and water use under fluctuating light. Here, we compared the photosynthetic and growth characteristics under constant and fluctuating light among three lines of *Arabidopsis thaliana* (L.): the wild type (WT), *STOMAGEN/EPFL9*-overexpressing line (ST-OX), and *EPIDERMAL PATTERNING FACTOR 1* knockout line (*epf1*). ST-OX and *epf1* showed 268.1 and 46.5% higher *SD* than WT (*p* < 0.05). Guard cell length of ST-OX was 10.0% lower than that of WT (*p* < 0.01). There were no significant variations in gas exchange parameters at steady state between WT and ST-OX or *epf1*, although these parameters tended to be higher in ST-OX and *epf1* than WT. On the other hand, ST-OX and *epf1* showed faster *A* induction than WT after step increase in light owing to the higher *g*_*s*_ under initial dark condition. In addition, ST-OX and *epf1* showed initially faster *g*_*s*_ induction and, at the later phase, slower *g*_*s*_ induction. Cumulative CO_2_ assimilation in ST-OX and *epf1* was 57.6 and 78.8% higher than WT attributable to faster *A* induction with reduction of water use efficiency (*WUE*). *epf1* yielded 25.6% higher biomass than WT under fluctuating light (*p* < 0.01). In the present study, higher *SD* resulted in faster photosynthetic induction owing to the higher initial *g*_*s*_. *epf1*, with a moderate increase in *SD*, achieved greater biomass production than WT under fluctuating light. These results suggest that higher *SD* can be beneficial to improve biomass production in plants under fluctuating light conditions.

## Introduction

Enhancing leaf photosynthesis has been attempted to drive further increases in biomass production in crop plants (von Caemmerer and Evans, 2010; Yamori et al., 2016; Sakoda et al., 2018). Gas diffusional resistance from the atmosphere to the chloroplast is one of the limiting factors for leaf photosynthetic capacity (Farquhar and Sharkey, 1982). Stomata, pores on the epidermis of plant leaves, function to maintain the balance between CO_2_ uptake for photosynthesis and water loss for transpiration (Mcadam and Brodribb, 2012). It has been highlighted that the conductance to gas diffusion via stomata (*g*_*s*_) can be a major determinant of CO_2_ assimilation rate (*A*) (Wong et al., 1979). The potential of *g*_*s*_ is mainly determined by the size, depth, and opening of single stoma, and their density (Franks and Beerling, 2009). It has been controversial how the change in the stomatal density (*SD*), defined as the stomata number per unit leaf area, affects photosynthetic and growth characteristics in plants (Lawson and Blatt, 2014). Doheny-Adams et al. (2012) reported that lower *SD* yielded higher growth rate and biomass production in Arabidopsis under constant light owing to the favorable water condition and temperature for metabolism and low metabolic cost for stomatal development (Doheny-Adams et al., 2012). Contrastingly, lower *SD* resulted in the depression of *g*_*s*_ and/or *A* in Arabidopsis and poplar plants (Büssis et al., 2006; Yoo et al., 2010; Wang et al., 2016). An *SDD1* knockout line of Arabidopsis with higher *SD* showed higher *g*_*s*_ and *A* than a wild-type line, depending on light condition (Schlüter et al., 2003). Previously, we reported that higher *SD* by overexpressing *STOMAGEN/EPFL9* resulted in the enhancement of *g*_*s*_ and *A* in Arabidopsis under constant and high light conditions (Tanaka et al., 2013). Therefore, *SD* manipulation could have the potential to enhance photosynthetic and growth characteristics in plants, even though that effect can depend on the species or environmental conditions.

In the field, light intensity can fluctuate at different scales, from less seconds to minutes, over the course of a day owing to changes in the solar radiation, cloud cover, or self-shading in the plant canopy (Kaiser et al., 2018). The gradual increase in *A* can be shown after the transition from low to high light intensity, and this phenomenon is called “photosynthetic induction.” A simulation analysis demonstrated that the potential loss of the cumulative amount of CO_2_ assimilation caused by photosynthetic induction can reach at least 21% in wheat (*Triticum aestivum* L.) and soybean (*Glycine max* (L.) Merr.) (Taylor and Long, 2017; Tanaka et al., 2019). In rice (*Oryza sativa* L.) and soybean, there is genotypic variation in the speed of photosynthetic induction, which causes significant differences in the cumulative carbon gain under fluctuating light (Soleh et al., 2016, 2017; Adachi et al., 2019). Consequently, speeding up photosynthetic induction can yield more efficient carbon gain, which will open a new pathway to improve biomass production in plants under field conditions.

Photosynthetic induction is typically limited by three phases of the biochemical and diffusional processes: (1) activation of electron transport, (2) activation of the enzymes in the Calvin-Benson cycle, and (3) stomatal opening (Pearcy, 1990; Yamori, 2016; Yamori et al., 2020). Especially, the activation of Rubisco (5–10 min for full induction) and stomatal opening (20–30 min for full induction) constitute a major limitation to photosynthetic induction (Yamori et al., 2012; Carmo-Silva and Salvucci, 2013). The overexpression of *PATROL1*, controlling the translocation of a major H^+^-ATPase (AHA1) to the plasma membrane, resulted in faster *g*_*s*_ induction to fluctuating light in Arabidopsis without the change in *SD* (Hashimoto-Sugimoto et al., 2013). Arabidopsis knockout mutants of ABA transporter, which plays pivotal roles in stomatal closure, improved stomatal response to fluctuating light and photosynthesis (Shimadzu et al., 2019). Furthermore, the rapid stomatal response is important for plants to achieve high water use efficiency (*WUE*) (Qu et al., 2016). Notably, the faster stomatal opening improved the photosynthetic induction and thus biomass production in Arabidopsis under the fluctuating light (Papanatsiou et al., 2019; Kimura et al., 2020). These facts evidence that rapid stomatal response can be beneficial for the effective carbon gain and water use under fluctuating light conditions. However, how *SD* changes affect *g*_*s*_ and *A* dynamics, biomass production, and water use under these conditions has been understudied (Drake et al., 2013; Papanatsiou et al., 2016; Schuler et al., 2017; Vialet-Chabrand et al., 2017).

It is hypothesized that higher *SD* results in higher initial *g*_*s*_ (Tanaka et al., 2013), which can contribute to faster photosynthetic induction due to the lower stomatal limitation under the fluctuating light. The objective of this study was to examine how higher *SD* affects the photosynthetic and growth characteristics in plants under fluctuating light conditions. Here, we investigated the induction response of *g*_*s*_, *A*, transpiration rate (*E*) and water use efficiency (*WUE*) after step increase in light by gas exchange measurements, and biomass production under fluctuating light conditions in the three Arabidopsis lines differing in *SD*.

## Materials and Methods

### Plant Materials and Growth Conditions

The peptide signals in a family of EPIDERMAL PATTERNING FACTOR (EPF) were identified to function in the stomatal development of Arabidopsis (*Arabidopsis thaliana* (L.) Heynh) (Hara et al., 2007). It has been demonstrated that EPF1 and EPF2 combine with the receptor-like protein, TOO MANY MOUTHS (TMM) and ERECTA family leucine-rich repeat-receptor-like kinases and, consequently, restrain a specific process in stomatal development. Contrastingly, STOMAGEN/EPFL9 combines with TMM competitively to EPF1 and EPF2, and promote stomatal development (Sugano et al., 2010; Lee et al., 2015). In the present study, Columbia-0 (CS60000) of *Arabidopsis thaliana* (L.) Heynh, was used as a wild-type line (WT). In addition, we used *STOMAGEN/EPFL9* overexpressing line (ST-OX10-3; ST-OX) which was used in Tanaka et al. (2013), and an *EPF1* knockout line (SALK_137549) (*epf1-1*; *epf1*) which was used in Sugano et al. (2010).

For analyzing photosynthetic and stomatal traits, six plants per line were sown and grown in the soil in the growth chamber at an air humidity of 60%, CO_2_ concentration of 400 μmol mol^–1^ and a photosynthetic photon flux density (PPFD) of 100 μmol photon m^–2^ s^–1^ for the gas exchange analysis. The day/night period was set to 8/16 h with a constant air temperature of 22°C. We randomly changed plant arrangement every 3–4 days during their growth period to avoid the spacing effects. For the biomass analysis, plants were sown and grown in the soil at an air temperature of 22°C and a PPFD of 120 μmol photon m^–2^ s^–1^ for 24 days after sowing with the day/night period of 8/16 h. Subsequently, four plants per line were subjected to constant and fluctuating light conditions, for 20 days with a day/night cycle of 12/12 h. During daytime, the light intensity in the constant light condition was changed from a PPFD of 60 μmol photon m^–2^ s^–1^ for 4 h to 500 μmol photon m^–2^ s^–1^ for 4 h, followed by 60 μmol photon m^–2^ s^–1^ for 4 h, while a PPFD of 60 μmol photon m^–2^ s^–1^ for 10 min after 500 μmol photon m^–2^ s^–1^ for 5 min was repeated for 12 h in the fluctuating light condition as described in Kimura et al. (2020). Plants were exposed to the same total amount of light intensity per day under both light conditions. We randomly changed plant arrangement every 3–4 days during their growth period to avoid the spacing effects. Dry weight of above ground biomass grown under each light condition was evaluated at 44 days after sowing.

### Evaluation of Stomatal Density, Size, and Clustering

The stomatal density (*SD*), size (*L*_*g*_), and clustering were evaluated in the leaves of the six plants per line at the same growth stage as the gas exchange measurements were conducted. We used the six leaves of the three plants in which gas exchange measurements were conducted and the other three plants. A section of the leaf (5 × 5 mm) was excised and immediately fixed in the solution (Ethanol : acetic acid = 9:1, v/v) overnight. The fixed tissues were cleared in chloral hydrate solution (chloral hydrate : glycerol : water = 8:1:2, w/v/v) overnight. The cleared tissues were stained with safranin-O solution (200 μg ml^–1^) for 30 min to 1 h. The abaxial side of the leaves was observed at a 200 × magnification using an optical microscope and six digital images (0.072 mm^2^) were obtained per leaf (CX31 and DP21, Olympus, Tokyo, Japan). We used imaging analysis software, ImageJ (NIH, Bethesda, MD, United States) to assess the stomatal number and guard cell length from the images. *SD* was calculated from the stomatal number per unit leaf area. *L*_*g*_, defined as guard cell length, of all the stomata (2–86 stomata) was measured in each image. Each clustering category (2–5 er) means the number of clustered stomata. The percentage of clustered stomata to total number was measured for each clustering category from 2 to 5 as described in Hara et al. (2007). The mean values of each trait were calculated in six images obtained from each leaf. Subsequently, the average value of each trait for six leaves was calculated for each line.

### Gas Exchange Measurements

Gas exchange measurements were conducted using a portable gas-exchange system LI-6400 (*LI-COR*, Lincoln, NE, United States). All plants were kept in the dark (a PPFD of 0 μmol photon m^–2^ s^–1^) overnight before and during the measurements. In the leaf chamber, we set flow rate at 300 μmol s^–1^, CO_2_ concentration at 400 μmol mol^–1^, and air temperature at 25°C. After the leaf was clamped in the chamber, light intensity was kept at a PPFD of 0 μmol photon m^–2^ s^–1^ for the initial 10 min and, subsequently, under a PPFD of 500 μmol photon m^–2^ s^–1^ for 120 min. *A*, *g*_*s*_, intercellular CO_2_ concentration (*C*_*i*_), and *E* were recorded every 10 s during the measurements. *WUE* was calculated as the ratio of *A* to *E*. Gas exchange measurements were conducted with three plants per line during 68 to 73 days after sowing.

### Data Processing

To evaluate the induction speeds of *A* and *g*_*s*_, we calculated *A*_*induction*_ and *g*_*s*__*induction*_ defined as the following equations:

(1) $$g_{s\,i\,n\,d\,u\,c\,t\,i\,o\,n}=\frac{g_{s\,t}-g_{s\,i}}{g_{s\,f}-g_{s\,i}}$$

(2) $$A_{i\,n\,d\,u\,c\,t\,i\,o\,n}=\frac{A_{t}-A_{i}}{A_{f}-A_{i}}$$

where *A*_*i*_ and *g*_*si*_ represent steady-state values under a PPFD of 0 μmol photon m^–2^ s^–1^, steady-state *A* and *g*_*s*_, *A*_*f*_ and *g*_*s*__*f*_, represent the maximum values which were reached in 120 min under a PPFD of 500 μmol photon m^–2^ s^–1^, and *A*_*t*_ and *g*_*s*_*_*t*_* represent values at a given time under illumination. We evaluated the differences in the time when *A*_*induction*_ and *g*_*sinduction*_ reached the closest values of 5, 10, 20, 40, 60, and 80% of those maximum values after step change in light from 0 to 500 μmol photon m^–2^ s^–1^ (*t*_5__–__80_*_*g*_*_*s*_ and *t*_5__–__80_*_*A*_*) between WT and ST-OX or *epf1*.

The cumulative CO_2_ assimilation (*CCA*) and transpiration (*CE*) under fluctuating light were calculated by summing *A* and *E* in first 10 min under illumination after the initial dark period. An integrated *WUE* (*WUE*_*i*_) was calculated as the ratio of *CCA* to *CE*. Assuming the absence of induction response of *A* to the step increase in light, a theoretically maximum *CCA* (*CCA*_*t*_) was defined by the following equation:

(3) $$C\,C\,A_{t}=A_{f}\cdot T_{500}$$

where *T*_500_ is the seconds for which the light intensity was maintained at 500 μmol photon m^–2^ s^–1^ for 10 min. The potential loss rate of *CCA* caused by photosynthetic induction was defined by the following equation:

(4) $$L\,o\,s\,s\,r\,a\,t\,e=\left(1-\frac{C\,C\,A}{C\,C\,A_{t}}\right)\times 100$$

### Statistical Analysis

The variation in stomatal size and all the parameters of photosynthetic and growth characteristics were compared between WT and ST-OX or *epf1* by a Dunnett’s test. Steel test was applied to evaluate *SD* variation between WT and ST-OX or *epf1* because the distribution of values was extremely different among the lines. Statistical analysis was conducted using R software version 3. 6. 1 (R Foundation for Statistical Computing, Vienna, Austria).

## Results

### Stomatal Density, Size, and Clustering

**FIGURE 1:**
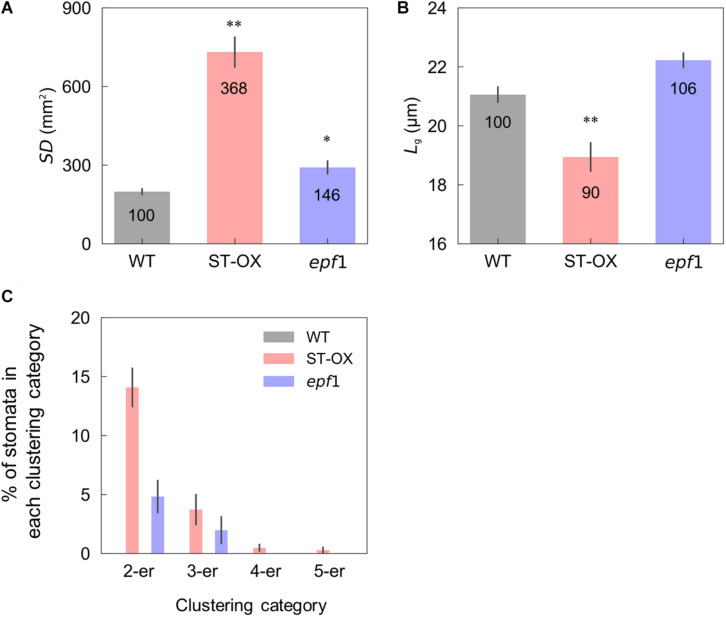
Stomatal density, size, and clustering. **(A)** The stomatal density (*SD*), **(B)** guard cell length (*L*_*g*_) and **(C)** the rate of stomata in 2–5 clustering categories were evaluated on fully expanded leaves in the wild-type line (WT), a *STOMAGEN*/*EPFL9* overexpressing line (ST-OX), and an *EPF1* knockout line (*epf1*) of *Arabidopsis thaliana*. The vertical bars indicate the standard error (*n* = 6). * and ** indicate the significant variation in each parameter between WT and each transgenic line at *p* < 0.05, and 0.01, respectively, according to the Steel test in **(A)** or Dunnett’s test in **(B)**. The value in each column represents the relative value of each line to WT.

We evaluated stomatal density (*SD*), size (*L*_*g*_) and clustering in the three Arabidopsis lines. ST-OX and *epf1* showed 268.1 and 46.5% higher *SD* than WT (*p* < 0.05) (Figure 1A). *L*_*g*_ of ST-OX was 10.0% lower than that of WT (*p* < 0.01) (Figure 1B). Stomatal clustering was scarcely observed in WT, while two to five stomata were clustered in ST-OX and *epf1* (Figure 1C). The ratio of stomata in each clustering category was higher in ST-OX than that in *epf1*.

### Photosynthesis and Stomatal Conductance After Step Increase in Light

**FIGURE 2:**
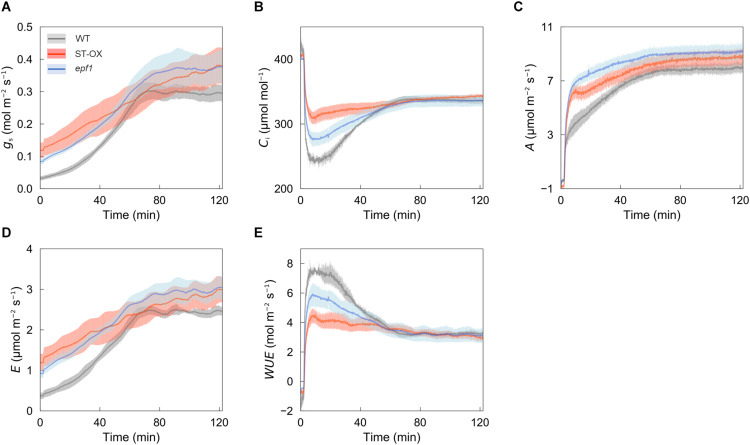
Photosynthetic dynamics after step increase in light. **(A)** A stomatal conductance (*g*_*s*_), **(B)** intercellular CO_2_ concentration (*C*_*i*_), **(C)** CO_2_ assimilation rate (*A*), **(D)** transpiration rate (*E*), and **(E)** water use efficiency (*WUE*) were measured on fully expanded leaves in the three lines of Arabidopsis. The gas exchange measurements were conducted at a CO_2_ concentration of 400 ppm, air temperature of 25°C and dark condition for the initial 10 min and, subsequently, under a PPFD of 500 μmol photon m^–2^ s^–1^ for 120 min. Vertical bars indicate the standard error (*n* = 3).

To examine how higher *SD* affects the photosynthetic characteristics under the fluctuating light, we conducted gas exchange measurements. ST-OX and *epf1* maintained higher *g*_*s*_, *C*_*i*_, *A*, and *E* than WT under the non-steady state at high light intensity (500 μmol photon m^–2^ s^–1^) (Figures 2A–D), while they showed lower *WUE* (Figure 2E). Under the steady state, there was no significant difference in *g*_*s*_ and *A* between WT and ST-OX or *epf1*, although these parameters of ST-OX and *epf1* tended to be higher than those of WT (Supplementary Figure S1).

**FIGURE 3:**
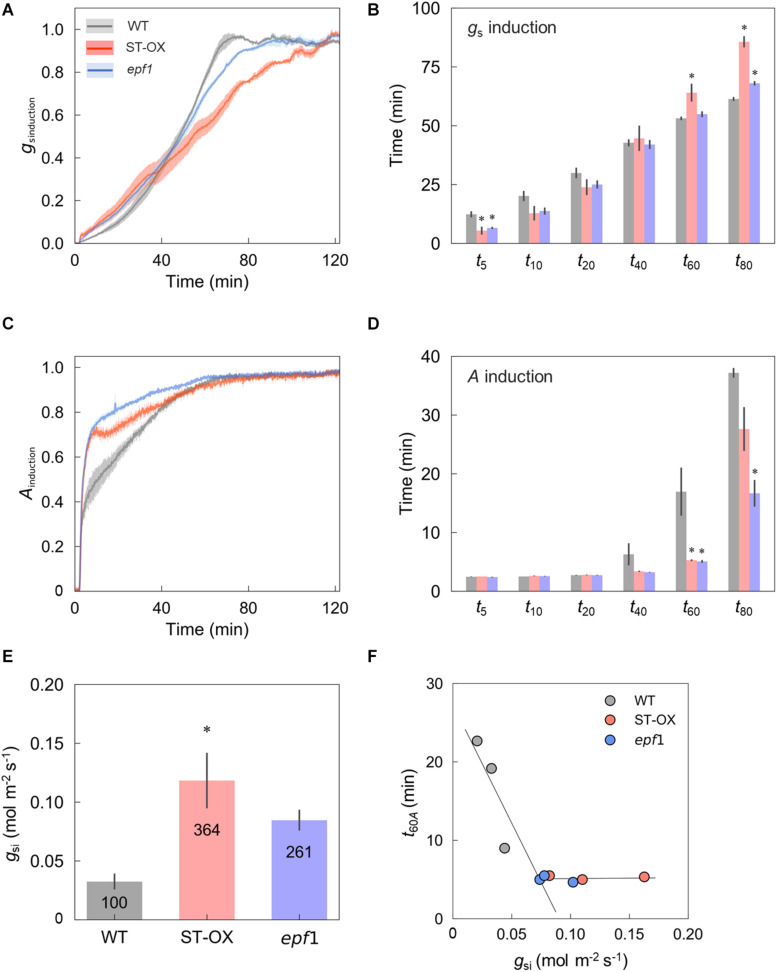
The induction speed of stomatal conductance and CO_2_ assimilation rate after step increase in light. The induction state of **(A)** stomatal conductance (*g*_*s*_) and **(C)** CO_2_ assimilation rate (*A*) were evaluated in the three lines of Arabidopsis based on *g*_*sinduction*_ and *A*_*induction*_ defined as Eqs. 1 and 2, respectively, under a PPFD of 500 μmol photon m^–2^ s^–1^ for 120 min after the dark period for 10 min. The time when **(B)** *g*_*s*__*induction*_ and **(D)** *A*_*induction*_ reached 5, 10, 20, 40, 60, and 80% (*t*_5__–__8__0_*_*g*_*_*s*_ and *t*_5__–__8__0_*_*A*_*) of those maximum values was compared between WT and each transgenic line. **(E)** The steady-state value of *g*_*s*_ under the dark condition (*g*_*s*__*i*_) was compared between WT and each transgenic line. **(F)** The relationship was investigated between *g*_*s*__*i*_ and *t*_60_*_*A*_*. Vertical bars indicate the standard error (*n* = 3). *indicates significant differences in each parameter between WT and each transgenic line at *p* < 0.05, according to Dunnett’s test. The values in each column represent the relative value of each line to WT.

Subsequently, we evaluated the induction speed of *g*_*s*_ and *A* to the step increase in light in the three Arabidopsis lines. After the change from darkness (0 μmol photon m^–2^ s^–1^) to high light, *g*_*s*_ induction was initially faster in ST-OX and *epf1* than WT during photosynthetic induction, while it was slower in ST-OX and *epf1* than WT at the later phase (Figure 3A). *g*_*s*_ in WT and *epf1* was fully induced at 80 min after step increase in light, while that of ST-OX slightly but continuously increased in 120 min (Figure 2A). *t*_5_*_*g*_*_*s*_ in ST-OX and *epf1* was significantly shorter than that in WT (*p* < 0.05) (Figure 3B). On the other hand, *t*_60_*_*g*_*_*s*_ in ST-OX and *t*_80_*_*g*_*_*s*_ in ST-OX and *epf1* were significantly larger than that in WT (*p* < 0.05). *A* induction was faster in ST-OX and *epf1* than WT after the step increase in light (Figure 3C). *t*_60_*_*A*_* in ST-OX and *epf1* and *t*_80_*_*A*_* in *epf1*were significantly shorter than that in WT (*p* < 0.05) (Figure 3D). In the steady state under darkness, *g*_*si*_ in ST-OX and *epf1* were 264.5% (*p* < 0.01) and 160.6% higher (not significant), respectively, than that in WT (Figure 3E). *t*_60_*_*A*_* decreased with the increase in *g*_*si*_ when *g*_*si*_ < 0.074, and it was constantly independent of *g*_*si*_ for *g*_*si*_ > 0.074 (Figure 3F).

### CO_2_ Assimilation and Biomass Production Under Fluctuating Light

**FIGURE 4:**
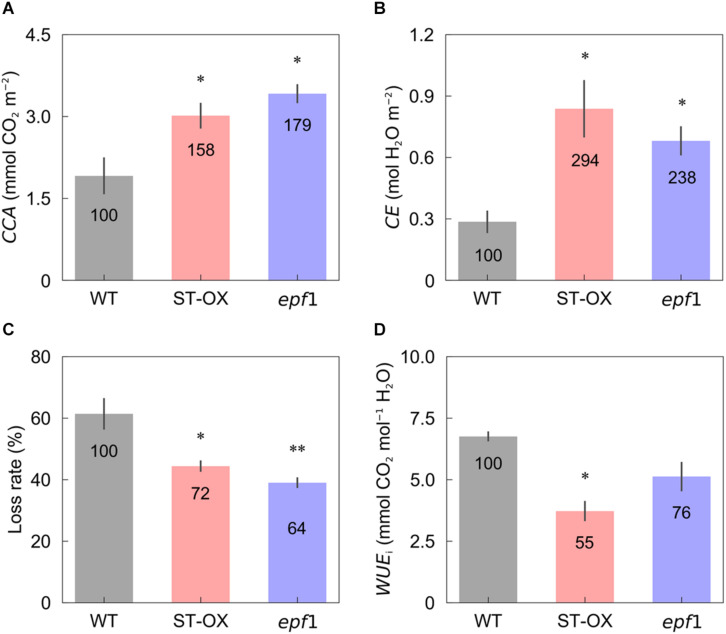
Cumulative carbon gain and water use after step increase in light. **(A)** Cumulative CO_2_ assimilation (*CCA*) and **(B)** transpiration (*CE*) were measured in the first 10 min under illumination after initial darkness in the three lines of Arabidopsis under the fluctuating light. **(C)** The loss rate of CO_2_ assimilation caused by the induction response was calculated based on Eq. 3. **(D)** Integrated water use efficiency (*WUE*_*i*_) was calculated as the ratio of *CCA* to *CE*. Vertical bars indicate the standard error (*n* = 3). * and ** indicate significant differences in each parameter between WT and each transgenic line at *p* < 0.05 and 0.01, respectively, according to Dunnett’s test. The value in each column represents the relative value of each line to WT.

**FIGURE 5:**
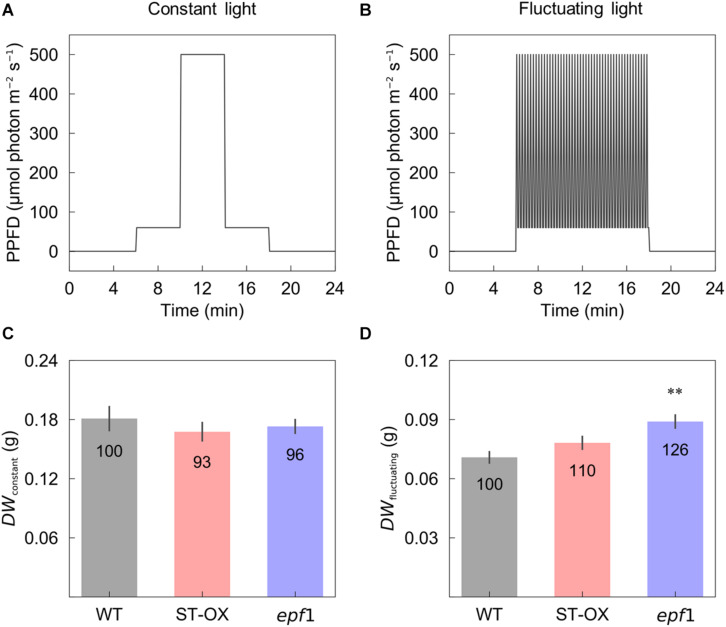
Biomass production under the constant and fluctuating light conditions. Dry weight of the above ground biomass was evaluated in the three lines of Arabidopsis under **(C)** the constant (*DW*_*constant*_) and **(D)** fluctuating (*DW*_*fluctuating*_) light conditions as described in **(A)** and **(B)**. Vertical bars indicate the standard error (*n* = 4). ** indicates significant differences in each parameter between WT and each transgenic line at *p* < 0.01, according to Dunnett’s test. The values in each column represent the relative value of each line to WT.

Cumulative CO_2_ assimilation and transpiration were evaluated to compare the efficiency of carbon gain and water use during photosynthetic induction in the three Arabidopsis lines. *CCA* in ST-OX and *epf1* was 57.6 and 78.8% higher (*p* < 0.05), respectively, than that in WT, while Loss rate in ST-OX and *epf1* was 27.7% and 36.5% lower (*p* < 0.05) (Figures 4A,C). *CE* in ST-OX and *epf1* were 193.7% and 138.7% higher (*p* < 0.05), respectively, than that in WT (Figure 4B). There was no significant variation in *WUE*_*i*_ between WT and *epf1*, while *WUE*_*i*_ in ST-OX was 44.9% lower than WT (*p* < 0.05) (Figure 4D). Finally, we evaluated the biomass production under the constant (Figure 5A) and fluctuating light (Figure 5B) in the three Arabidopsis lines to examine how higher *SD* affects growth characteristics. Compared with WT, dry weight of the above ground biomass under constant light (*DW*_*constant*_) in *epf1* was similar, while that under fluctuating light (*DW*_*fluctuating*_) in *epf1* was 25.6% higher than that of WT (*p* < 0.01) (Figures 5C,D). There was no significant variation in *DW*_*constant*_ and *DW*_*fluctuating*_ between ST-OX and WT.

## Discussion

Stomata play a significant role in the regulation of gas exchange between the outside and inside of the leaf. However, how the *SD* change affects photosynthetic and growth characteristics in plants has been controversial, and the effect of *SD* change on photosynthesis and growth can vary depending on the plant species or environmental conditions. Previously, we reported that higher *SD* resulted in the enhancement of *g*_*s*_ and *A* in Arabidopsis under constant and saturated light conditions (Tanaka et al., 2013). Lawson and Blatt (2014) suggested that with higher *SD*, it would be instructive to determine biomass productivity under fluctuating light, although only a few studies investigated the relationship between *SD* and photosynthetic or growth characteristics under that condition (Drake et al., 2013; Papanatsiou et al., 2016; Schuler et al., 2017; Vialet-Chabrand et al., 2017). Here, we attempted to examine how higher *SD* affects *g*_*s*_ and *A* dynamics, biomass production, and water use in Arabidopsis under fluctuating light.

### Stomatal Density Affects the Induction of Stomatal Opening

We revealed that the three Arabidopsis lines differing in *SD* showed significant differences in the dynamics of *g*_*s*_ in the non-steady state. *SD* differences had significant (Vialet-Chabrand et al., 2017) or non-significant (Papanatsiou et al., 2016; Schuler et al., 2017) effect on *g*_*s*_ induction to light transients from low to high in previous studies. In the present study, ST-OX and *epf1* showed initially faster *g*_*s*_ induction than WT, while those lines showed slower *g*_*s*_ induction in the later phase after step increase in light from a PPFD of 0 to 500 μmol photon m^–2^ s^–1^ (Figures 3A,B). The different responses of *g*_*s*_ could be attributable to the difference in the size, density, and patterning of stomata. Drake et al. (2013) reported that smaller stomata respond the fluctuating light faster than larger stomata among several species of the genus *Banksia*. On the contrary, smaller stomata resulted in the slower response of *g*_*s*_ to fluctuating light in the genus *Oryza* (Zhang et al., 2019). In the present study, the variation in the speed of *g*_*s*_ induction did not correspond to that in *L*_*g*_ (Figures 1, 3), indicating that the stomatal size would have a minor effect on *g*_*s*_ induction in Arabidopsis under fluctuating light.

The stomatal opening is regulated by at least three key components, blue-light receptor phototropin, plasma membrane H^+^-ATPase, and plasma membrane inward rectifying K^+^ channels in the guard cell (Inoue and Kinoshita, 2017). The activation of H^+^-ATPase induced by blue light as the initial signal facilitates K^+^ uptake through the inward rectifying K^+^ channel to increase the turgor pressure of guard cells, resulting in the stomatal opening. In addition, stomatal opening dynamics depend on the water status in the plant (Lawson and Blatt, 2014). With more stomata, higher metabolic cost and water uptake would be required for stomatal movement. The gas-exchange and theoretical-modeling analysis indicated that the stomatal clustering decreased the maximum value of *g*_*s*_ and *A* under the steady state because of the misplacement of stomatal pores over mesophyll cells (Dow and Bergmann, 2014; Lehmann and Or, 2015). It was also shown that clustering suppressed stomatal movement owing to the decreased capacity of the K^+^ flux and K^+^ accumulation in the guard cells (Papanatsiou et al., 2016). Additionally, *g*_*s*_ induction to fluctuating light in *Begonia* species with clustered stomata was slower than that in those without clustered stomata (Papanatsiou et al., 2017). In this study, ST-OX and *epf1* with higher *SD* and clustering rate showed initially faster *g*_*s*_ induction and, at the later phase, slower induction than WT (Figures 1, 3). These results suggest that the changes in stomatal density and patterning can affect *g*_*s*_ induction to fluctuating light owing to the change in water uptake for stomatal opening and the opening speed of single stomata.

### Stomatal Density Affects the Dynamics of CO_2_ Assimilation

In ST-OX and *epf1*, *A* induction to step change from darkness to high light was faster than that of WT (Figures 3C,D). Photosynthetic induction is typically limited by three phases of the biochemical or diffusional processes; (1) activation of electron transport, (2) activation of the enzymes of the Calvin-Benson cycle, and (3) stomatal opening (Pearcy, 1990; Yamori et al., 2016). The significance of stomatal limitation to photosynthetic induction depends on the initial value of *g*_*s*_ as well as photosynthetic capacity and the induction state of biochemical processes (Kirschbaum and Pearcy, 1988). Activation speed of the electron transport and enzymes of the Calvin-Benson cycle after step increase in light intensity can be largely affected by CO_2_ concentration (Jackson et al., 1991; Urban et al., 2008; Kaiser et al., 2017). The variation of *g*_*s*_ under dark or low light conditions corresponded to that in the speed of photosynthetic induction in several plant species (Kaiser et al., 2016; Soleh et al., 2017). In this study, *t*_60_*_*A*_* correlated with *g*_*si*_ if *g*_*si*_< 0.074 mol m^–2^ s^–1^, and it was constant regardless of *g*_*si*_ if *g*_*si*_> 0.074 mol m^–2^ s^–1^ (Figure 3F). *g*_*s*__*i*_ of WT, ST-OX, and *epf1* were 0.032, 0.118, and 0.085 mol m^–2^ s^–1^, respectively (Figure 3E), suggesting that the variation in *g*_*s*__*i*_ would cause the response difference of *A*. Therefore, higher *SD* resulted in higher initial value of *g*_*s*_ and then higher *C*_*i*_, which would contribute to the rapid activation of RuBP regeneration and carboxylation in the Calvin-Benson cycle.

The transition from a short period of low to high light is frequently observed in the crop canopy throughout the day (Tanaka et al., 2019). The present study confirmed that higher *SD* resulted in faster *A* induction after step increase in light from darkness, which can be observed at the limited part of the day in field. It is not clear how *SD* affects *g*_*s*_ and *A* induction after the adaptation to low light for short period. It has been considered that stomatal opening and Rubisco activation would not be a major limitation to *A* under such light conditions since these would not change rapidly (MuAusland et al., 2016). A rapid change in the RuBP regeneration was reported to limit photosynthetic induction under high light after a short period of low light or darkness (Kobza and Edwards, 1987; Sassenrath-Cole and Pearcy, 1994). On the other hand, the significant stomatal limitation to photosynthesis has been shown in Arabidopsis (Kimura et al., 2020) and rice (Yamori et al., 2020) under natural light conditions where the light fluctuations are highly variable. Future study is required to elucidate that higher *SD* would be beneficial for carbon gain under more rapid and frequent fluctuation of light.

### Stomatal Density Affects Biomass Production Under the Fluctuating Light

Manipulating CO_2_ diffusion via stomata has been attempted to enhance photosynthetic capacity and induction in plants. Under constant light conditions, overexpression of H^+^-ATPase (AHA2) in guard cells resulted in higher *g*_*s*_ as well as *A*, leading to greater biomass production in Arabidopsis (Wang et al., 2014). In addition, Arabidopsis plants with stay-opening or fast-moving stomata have been shown to achieve greater carbon gain and biomass production under fluctuating light conditions (Papanatsiou et al., 2019; Kimura et al., 2020). These studies confirmed the significant limitation of photosynthesis imposed by stomata, and the potential of *g*_*s*_ to improve biomass production of plants under field. On the other hand, higher *g*_*s*_ generally results in lower *WUE*, which can depress the benefit of greater photosynthetic performance for biomass production (Tanaka et al., 2013; Kimura et al., 2020). Under drought condition, transgenic plants with lower *SD* and *g*_*s*_ exhibited improved growth performance owing to high *WUE* in several species (Yoo et al., 2010; Wang et al., 2016; Caine et al., 2018). It is, therefore, import to optimize a balance between carbon gain and water loss via stomata for plant growth depending on water conditions (Lawson and Blatt, 2014; MuAusland et al., 2016).

*DW*_*fluctuating*_ was much lower than *DW*_*constant*_ in three Arabidopsis lines, although the total amount of light intensity exposed to the plants was equal between both light conditions (Figure 5). This difference would be caused by the loss of carbon gain owing to photosynthetic induction under fluctuating light condition. *DW*_*constant*_ in ST-OX was slightly lower than that in WT, although steady-state *A* was significantly or slightly higher in Tanaka et al. (2013) and this study, respectively (Figures 2, 5). The increase in water loss would have a negative effect on biomass production in ST-OX under constant light (Tanaka et al., 2013). ST-OX showed significantly lower *WUE* during photosynthetic induction in the present study (Figures 2, 4). Despite of these penalties resulting from the drastic increase in *SD*, *DW*_*fluctuating*_ in ST-OX was 10.5% higher than that in WT with no significance. Moreover, biomass production in *epf1*, with moderate increase in *SD*, was significantly higher than that in WT under fluctuating light, while there was no difference between these two lines under constant light (Figure 5). It is possible that a moderate increase in *SD* could achieve more efficient carbon gain attributable to the faster response of *A* in Arabidopsis under fluctuating light, while it would cause small penalties on water loss for stomatal movement. Overall, higher *SD* can be beneficial to improve biomass production in plants under fluctuating light conditions under favorable water conditions.

## Conclusion

Under fluctuating light, there was a significant variation in the photosynthetic and growth characteristics among Arabidopsis lines differing in the stomatal density (*SD*). Higher *SD* resulted in faster CO_2_ assimilation rate (*A*) induction to fluctuating light owing to the higher initial value of the stomatal conductance (*g*_*s*_) and faster *g*_*s*_ induction in the early phase of photosynthetic induction. On the other hand, higher *SD* resulted in slower *g*_*s*_ induction in the later phase of photosynthetic induction. *epf1*, with a moderate increase in *SD*, achieved more efficient carbon gain with small penalty on water use efficiency attributable to the faster *A* induction, which would contribute to higher biomass production than that in WT under fluctuating light. This study suggests that higher *SD* can be beneficial to improve biomass production in plants under fluctuating light.

## Data Availability Statement

The original contributions presented in the study are included in the article/Supplementary Material, further inquiries can be directed to the corresponding author.

## Author Contributions

KS conceived and designed this project, performed all the gas exchange experiments, wrote the manuscript with inputs from co-authors. WY conducted the biomass analysis. All authors contributed to the article and approved the submitted version.

## Conflict of Interest

The authors declare that the research was conducted in the absence of any commercial or financial relationships that could be construed as a potential conflict of interest.

## Supplementary Material

Supplementary Figure 1 Stomatal conductance and CO2 assimilation rate under steady state. **(A)** A stomatal conductance (*g*_*sf*_) and **(B)** CO_2_ assimilation rate (*A*_*f*_) under steady state were measured on fully expanded leaves in the three lines of Arabidopsis. The gas exchange measurements were conducted at a CO_2_ concentration of 400 ppm, air temperature of 25°C and dark condition for the initial 10 min and, subsequently, under a PPFD of 500 μmol photon m^–2^ s^–1^ for 120 min. Vertical bars indicate the standard error (*n* = 3). The values in each column represent the relative value of each line to WT.

## References

[B1] Adachi S. Tanaka Y. Miyagi A. Kashima M. Tezuka A. Toya Y. (2019). High-yielding rice Takanari has superior photosynthetic response under fluctuating light to a commercial rice *Koshihikari*. *J. Exp. Bot.* 70 5287–5297. 10.1093/jxb/erz304 31257443 PMC6793460

[B2] Büssis D. Von Groll U. Fisahn J. Altmann T. (2006). Stomatal aperture can compensate altered stomatal density in *Arabidopsis thaliana* at growth light conditions. *Funct. Plant Biol.* 33 1037–1043. 10.1071/FP06078 32689314

[B3] Caine R. Yin X. Sloan J. Harrison E. L. Mohammed U. Fulton T. (2018). Rice with reduced stomatal density conserves water and has improved drought tolerance under future climate conditions. *New Phytol.* 221 371–384. 10.1111/nph.15344 30043395 PMC6492113

[B4] Carmo-Silva A. E. Salvucci M. E. (2013). The regulatory properties of rubisco activase differ among species and affect photosynthetic induction during light transitions. *Plant Physiol.* 161 1645–1655. 10.1104/pp.112.213348 23417088 PMC3613445

[B5] Doheny-Adams T. Hunt L. Franks P. J. Beerling D. J. Gray J. E. (2012). Genetic manipulation of stomatal density influences stomatal size, plant growth and tolerance to restricted water supply across a growth carbon dioxide gradient. *Philos. Trans. R. Soc. B Biol. Sci.* 367 547–555. 10.1098/rstb.2011.0272 22232766 PMC3248714

[B6] Dow G. J. Bergmann D. C. (2014). Patterning and processes: how stomatal development defines physiological potential. *Curr. Opin. Plant Biol.* 21 67–74. 10.1016/j.pbi.2014.06.007 25058395

[B7] Drake P. L. Froend R. H. Franks P. J. (2013). Smaller, faster stomata: Scaling of stomatal size, rate of response, and stomatal conductance. *J. Exp. Bot.* 64 495–505. 10.1093/jxb/ers347 23264516 PMC3542046

[B8] Farquhar G. D. Sharkey T. D. (1982). Stomatal conductance and photosynthesis. *Annu. Rev. Plant Physiol.* 33 317–345. 10.1146/annurev.pp.33.060182.001533

[B9] Franks P. J. Beerling D. J. (2009). CO2-forced evolution of plant gas exchange capacity and water-use efficiency over the phanerozoic. *Geobiology* 7 227–236. 10.1111/j.1472-4669.2009.00193.x 19338614

[B10] Hara K. Kajita R. Torii K. U. Bergmann D. C. Kakimoto T. (2007). The secretory peptide gene EPF1. *Genes Dev.* 7 1720–1725. 10.1101/gad.1550707.metric PMC1920166 17639078

[B11] Hashimoto-Sugimoto M. Higaki T. Yaeno T. Nagami A. Irie M. Fujimi M. (2013). A Munc13-like protein in Arabidopsis mediates H+-ATPase translocation that is essential for stomatal responses. *Nat. Commun.* 4:3215. 10.1038/ncomms3215 23896897 PMC3731666

[B12] Inoue S. Kinoshita T. (2017). Blue light regulation of stomatal opening and the plasma membrane H ^+^ -ATPase. *Plant Physiol.* 174 531–538. 10.1104/pp.17.00166 28465463 PMC5462062

[B13] Jackson R. B. Woodrow I. E. Mott K. A. (1991). Nonsteady-state photosynthesis following an increase in photon flux density (PFD). *Plant Physiol*. 95 498–503. 10.1104/pp.95.2.498 16668012 PMC1077559

[B14] Kaiser E. Kromdijk J. Harbinson J. Heuvelink E. Marcelis L. F. M. (2017). Photosynthetic induction and its diffusional, carboxylation and electron transport processes as affected by CO_2_ partial pressure, temperature, air humidity and blue irradiance. *Ann. Bot.* 119 191–205. 10.1093/aob/mcw226 28025286 PMC5218377

[B15] Kaiser E. Morales A. Harbinson J. (2018). Fluctuating light takes crop photosynthesis on a rollercoaster ride. *Plant Physiol.* 176 977–989. 10.1104/pp.17.01250 29046421 PMC5813579

[B16] Kaiser E. Morales A. Harbinson J. Heuvelink E. Prinzenberg A. E. Marcelis L. F. M. (2016). Metabolic and diffusional limitations of photosynthesis in fluctuating irradiance in Arabidopsis thaliana. *Sci. Rep.* 6 1–13. 10.1038/srep31252 27502328 PMC4977489

[B17] Kimura H. Hashimoto-Sugimoto M. Iba K. Terashima I. Yamori W. (2020). Improved stomatal opening enhances photosynthetic rate and biomass production in fluctuating light. *J. Exp. Bot.* 71 2339–2350. 10.1093/jxb/eraa090 32095822

[B18] Kirschbaum M. U. F. Pearcy R. W. (1988). Gas exchange analysis of the fast phase of photosynthetic induction in *Alocasia macrorrhiza*. *Plant Physiol.* 87 818–821. 10.1104/pp.87.4.818 16666231 PMC1054852

[B19] Kobza J. Edwards G. E. (1987). The photosynthetic induction response in wheat leaves: net CO_2_ uptake, enzyme activation, and leaf metabolites. *Planta* 171 549–559. 10.1007/BF00392305 24225719

[B20] Lawson T. Blatt M. R. (2014). Stomatal size, speed, and responsiveness impact on photosynthesis and water use efficiency. *Plant Physiol.* 164 1556–1570. 10.1104/pp.114.237107 24578506 PMC3982722

[B21] Lee J. S. Hnilova M. Maes M. Lin Y. C. L. Putarjunan A. Han S. K. (2015). Competitive binding of antagonistic peptides fine-tunes stomatal patterning. *Nature* 522 439–443. 10.1038/nature14561 26083750 PMC4532310

[B22] Lehmann P. Or D. (2015). Effects of stomatal clustering on leaf gas exchange. *New Phytol.* 15 1015–1025. 10.1111/nph.13442 25967110

[B23] Mcadam S. A. M. Brodribb T. J. (2012). Stomatal innovation and the rise of seed plants. *Ecol. Lett.* 15 1–8. 10.1111/j.1461-0248.2011.01700.x 22017636

[B24] MuAusland L. Vialet-Chanbrand S. Davey P. Baker N. R. Brendel O. Lawson T. (2016). Effects of kinetics of light-induced stomatal responses on photosynthesis and water-use efficiency. *New Phytol.* 211 1209–1220. 10.1111/nph.14000 27214387 PMC4982059

[B25] Papanatsiou M. Amtmann A. Blatt M. R. (2016). Stomatal spacing safeguards stomatal dynamics by facilitating guard cell ion transport independent of the epidermal solute reservoir. *Plant Physiol.* 172 254–263. 10.1104/pp.16.00850 27406168 PMC5074606

[B26] Papanatsiou M. Amtmann A. Blatt M. R. (2017). Stomatal clustering in Begonia associates with the kinetics of leaf gaseous exchange and influences water use efficiency. *J. Exp. Bot.* 68 2309–2315. 10.1093/jxb/erx072 28369641 PMC5447881

[B27] Papanatsiou M. Petersen J. Henderson L. Wang Y. Christie J. M. Blatt M. R. (2019). Optogenetic manipulation of stomatal kinetics improves carbon assimilation, water use, and growth. *Science* 363 1456–1459. 10.1126/science.aaw0046 30923223

[B28] Pearcy R. W. (1990). Sunflecks and photosynthesis in plant canopies. *Ann. Rev. Plant Biol.* 41 421–453. 10.1146/annurev.pp.41.060190.002225

[B29] Qu M. Hamdani S. Li W. Wang S. Tang J. Chen Z. (2016). Rapid stomatal response to fluctuating light: an under-explored mechanism to improve drought tolerance in rice. *Funct. Plant Biol.* 43:727. 10.1071/fp15348 32480499

[B30] Sakoda K. Kaga K. Tanaka Y. Suzuki S. Fujii K. Ishimoto M. (2018). Two novel quantitative trait loci affecting the variation in leaf photosynthetic capacity among soybeans. *Plant Sci.* 291:110300. 10.1016/j.plantsci.2019.110300 31928682

[B31] Sakoda K. Yamori W. Shimada T. Sugano S. S. Hara-Nishimura I. Tanaka Y. (2020). Stomatal density affects gas diffusion and CO_2_ assimilation dynamics in *Arabidopsis* under fluctuating light. *bioRxiv* [Preprint]. 10.1101/2020.02.20.958603 PMC7641607 33193542

[B32] Sassenrath-Cole G. F. Pearcy R. W. (1994). Regulation of photosynthetic induction state by the magnitude and duration of low light exposure. *Plant Physiol.* 105 1115–1123. 10.1016/j.biopsych.2016.03.2100 12232269 PMC159439

[B33] Schlüter U. Muschak M. Berger D. Altmann T. (2003). Photosynthetic performance of an *Arabidopsis* mutant with elevated stomatal density (sdd1-1) under different light regimes. *J. Exp. Bot.* 54 867–874. 10.1093/jxb/erg087 12554730

[B34] Schuler M. L. Sedelnikova O. V. Walker B. J. Westhoff P. Langdale J. A. (2017). SHORTROOT-mediated increase in stomatal density has no impact on photosynthetic efficiency. *Plant Physiol.* 176 752–772. 10.1104/pp.17.01005 29127261 PMC5761779

[B35] Shimadzu S. Seo M. Terashima I. Yamori W. (2019). Whole irradiated plant leaves showed faster photosynthetic induction than individually irradiated leaves via improved stomatal opening. *Front. Plant Sci.* 10:1512. 10.3389/fpls.2019.01512 31850018 PMC6892984

[B36] Soleh M. A. Tanaka Y. Kim S. Y. Huber S. C. Sakoda K. Shiraiwa T. (2017). Identification of large variation in the photosynthetic induction response among 37 soybean [Glycine max (L.) Merr.] genotypes that is not correlated with steady-state photosynthetic capacity. *Photosynth. Res.* 131 305–315. 10.1007/s11120-016-0323-1 27878416

[B37] Soleh M. A. Tanaka Y. Nomoto Y. Iwahashi Y. Nakashima K. Fukuda Y. (2016). Factors underlying genotypic differences in the induction of photosynthesis in soybean [Glycine max (L.) Merr.]. *Plant Cell Environ.* 39 685–693. 10.1111/pce.12674 26538465

[B38] Sugano S. S. Shimada T. Imai Y. Okawa K. Tamai A. Mori M. (2010). Stomagen positively regulates stomatal density in *Arabidopsis*. *Nature* 463 241–244. 10.1038/nature08682 20010603

[B39] Tanaka Y. Adachi S. Yamori W. (2019). Natural genetic variation of the photosynthetic induction response to fluctuating light environment. *Curr. Opin. Plant Biol.* 49 52–59. 10.1016/j.pbi.2019.04.010 31202005

[B40] Tanaka Y. Sugano S. S. Shimada T. Hara-Nishimura I. (2013). Enhancement of leaf photosynthetic capacity through increased stomatal density in *Arabidopsis*. *New Phytol.* 198 757–764. 10.1111/nph.12186 23432385

[B41] Taylor S. H. Long S. P. (2017). Slow induction of photosynthesis on shade to sun transitions in wheat may cost at least 21% of productivity. *Philos. Trans. R. Soc. B Biol. Sci.* 17:372. 10.1098/rstb.2016.0543 28808109 PMC5566890

[B42] Urban O. Šprtová M. Košvancová M. Tomášková I. Lichtenthaler H. K. Marek M. V. (2008). Comparison of photosynthetic induction and transient limitations during the induction phase in young and mature leaves from three poplar clones. *Tree Physiol*. 28 1189–1197. 10.1093/treephys/28.8.1189 18519250

[B43] Vialet-Chabrand S. R. M. Matthews J. S. A. McAusland L. Blatt M. R. Griffiths H. Lawson T. (2017). Temporal dynamics of stomatal behavior: modeling and implications for photosynthesis and water use. *Plant Physiol.* 174 603–613. 10.1104/pp.17.00125 28363993 PMC5462030

[B44] von Caemmerer S. Evans J. R. (2010). Enhancing C3 photosynthesis. *Plant Physiol.* 154 589–592. 10.1104/pp.110.160952 20921190 PMC2948981

[B45] Wang C. Liu S. Dong Y. Zhao Y. Geng A. Xia X. (2016). PdEPF1 regulates water-use efficiency and drought tolerance by modulating stomatal density in poplar. *Plant Biotechnol. J.* 14 849–860. 10.1111/pbi.12434 26228739 PMC11388919

[B46] Wang Y. Noguchi K. Ono N. Inoue S. Terashima I. Kinoshita T. (2014). Overexpression of plasma membrane H^+^-ATPase in guard cells promotes light-induced stomatal opening and enhances plant growth. *Proc. Natl. Acad. Sci. U.S.A.* 111 533–538. 10.1073/pnas.1305438111 24367097 PMC3890815

[B47] Wong S. C. Cowan I. R. Farquhar G. D. (1979). Stomatal conductance correlates with photosynthetic capacity. *Nature* 282 424–426. 10.1038/282424a0

[B48] Yamori W. (2016). Photosynthetic response to fluctuating environments and photoprotective strategies under abiotic stress. *J. Plant Res.* 129 379–395. 10.1007/s10265-016-0816-1 27023791

[B49] Yamori W. Kondo E. Sugiura D. Terashima I. Suzuki Y. Makino A. (2016). Enhanced leaf photosynthesis as a target to increase grain yield: Insights from transgenic rice lines with variable Rieske FeS protein content in the cytochrome b6/f complex. *Plant Cell Environ.* 39 80–87. 10.1111/pce.12594 26138548

[B50] Yamori W. Kusumi K. Iba K. Terashima I. (2020). Increased stomatal conductance induces rapid changes to photosynthetic rate in response to naturally fluctuating light conditions in rice. *Plant. Cell Environ.* 43 1230–1240. 10.1111/pce.13725 31990076

[B51] Yamori W. Masumoto C. Fukayama H. Makino A. (2012). Rubisco activase is a key regulator of non-steady-state photosynthesis at any leaf temperature and, to a lesser extent, of steady-state photosynthesis at high temperature. *Plant J.* 71 871–880. 10.1111/j.1365-313X.2012.05041.x 22563799

[B52] Yoo C. Y. Pence H. E. Jin J. B. Miura K. Gosney M. J. Hasegawa P. M. (2010). The Arabidopsis GTL1 transcription factor regulates water use efficiency and drought tolerance by modulating stomatal density via transrepression of SDD1. *Plant Cell* 22 4128–4141. 10.1105/tpc.110.078691 21169508 PMC3027182

[B53] Zhang Q. Peng S. Li Y. (2019). Increase rate of light-induced stomatal conductance is related to stomatal size in the genus Oryza. *J. Exp. Bot.* 70 5259–5269. 10.1093/jxb/erz267 31145797 PMC6793446

