# Supplementary figures and images for: Higher Stomatal Density Improves Photosynthetic Induction and Biomass Production in Arabidopsis Under Fluctuating Light
Source: Front Plant Sci. 2020 Oct 21;11:589603. doi: 10.3389/fpls.2020.589603 (PMC7641607; doi:10.3389/fpls.2020.589603)

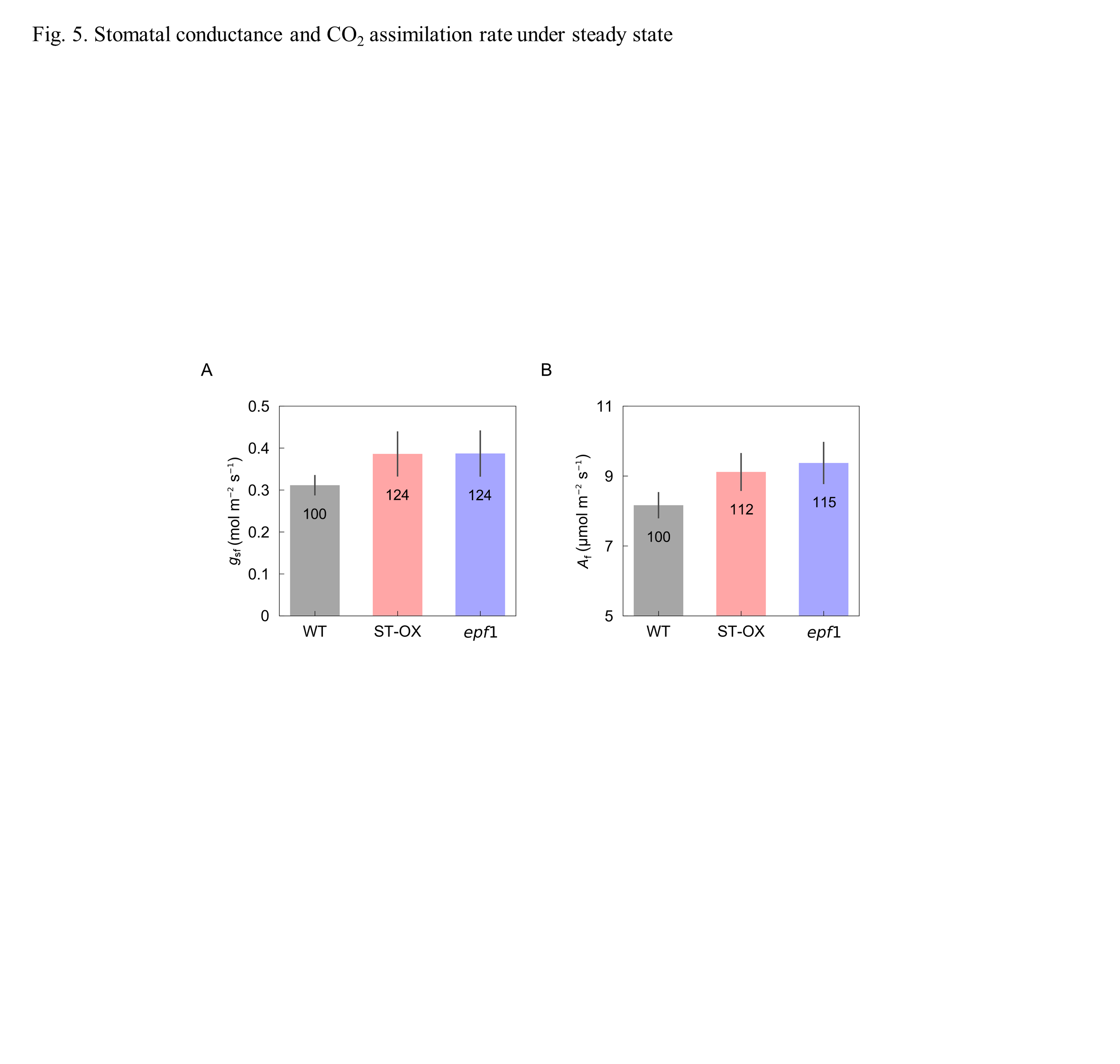

Supplement: Supplementary Figure 1 — Stomatal conductance and CO2 assimilation rate under steady state. (A) A stomatal conductance (gsf) and (B) CO2 assimilation rate (Af) under steady state were measured on fully expanded leaves in the three lines of Arabidopsis. The gas exchange measurements were conducted at a CO2 concentration of 400 ppm, air temperature of 25°C and dark condition for the initial 10 min and, subsequently, under a PPFD of 500 μmol photon m–2 s–1 for 120 min. Vertical bars indicate the standard error (n = 3). The values in each column represent the relative value of each line to WT. [file Image_1.TIF]
